# Angiotensin II Type 1 Receptor Antibodies Are Higher in Lupus Nephritis and Vasculitis than Other Glomerulonephritis Patients

**DOI:** 10.1007/s00005-022-00660-x

**Published:** 2022-09-24

**Authors:** Maciej Szymczak, Harald Heidecke, Marcelina Żabińska, Dagna Rukasz, Krzysztof Wiśnicki, Andrzej Tukiendorf, Magdalena Krajewska, Mirosław Banasik

**Affiliations:** 1 Department of Nephrology and Transplantation Medicine, Wroclaw Medical University, Wroclaw, Poland; 2 CellTrend Gmbh, Luckenwalde, Germany; 3 Department of Social Medicine, Wroclaw Medical University, Wroclaw, Poland

**Keywords:** Angiotensin II type 1 receptor antibodies, Lupus nephritis, Vasculitis, Glomerulonephritis

## Abstract

Angiotensin II type 1 receptor (AT1R) antibodies are considered non-HLA (human leukocyte antigen) antibodies connected with humoral rejection after kidney transplantation. The role of AT1R antibodies in the pathogenesis of glomerular diseases and systemic vasculitis is unknown. We assessed the level of AT1R antibodies in 136 patients with different types of glomerulonephritis and systemic vasculitis and we observed kidney function and proteinuria, serum albumin and total protein levels for 2 years. The mean levels of AT1R antibodies were the following: 6.00 ± 1.31 U/ml in patients with membranous nephropathy (*n* = 18), 5.67 ± 1.31 U/ml with focal and segmental glomerulosclerosis (*n* = 25), 6.26 ± 2.25 U/ml with lupus nephropathy (*n* = 17), 10.60 ± 6.72 U/ml with IgA nephropathy (*n* = 14), 6.69 ± 2.52 U/ml with mesangial proliferative (non IgA) glomerulonephritis (*n* = 6), 6.63 ± 1.38 U/ml with systemic vasculitis (*n* = 56), including c-ANCA (anti-neutrophil cytoplasmic antibodies) vasculitis: 11.22 ± 10.78 U/ml (*n* = 40) and p-ANCA vasculitis: 12.65 ± 14.59 U/ml (*n* = 16). The mean AT1R antibodies level was higher in patients with lupus nephropathy and systemic vasculitis compared to glomerulonephritis groups. An inverse statistically significant correlation between AT1R antibodies and serum albumin (*r* =  − 0.51) in membranous nephropathy group was also found. Prospective analysis of creatinine levels indicated an increase of creatinine levels during time among patients with higher AT1R antibodies levels in p-ANCA vasculitis. Lupus nephropathy and systemic vasculitis patients may have high levels of AT1R antibodies. AT1R antibodies may be associated with the severity of membranous nephropathy and the course of p-ANCA vasculitis, although influence of concomitant factors is difficult to exclude.

## Introduction

Angiotensin II type 1 receptor (AT1R) antibodies are connected with the rejection of renal allografts in the absence of human leukocyte antigen (HLA) antibodies (Dragun et al. 2005). Patients after kidney transplantation have a higher risk of kidney graft failure in the case of AT1R antibody presence (Taniguchi et al. 2012). The presence of these antibodies has a negative influence on renal transplant function in the early period after transplantation (Banasik et al. 2014a). High levels of AT1R antibodies (more than 9 U/l) increase graft loss risk (Banasik et al. 2014b). AT1R antibodies associated with humoral rejection have a specific clinical phenotype with high levels of endothelial-associated transcripts, lack of complement deposition in allograft capillaries, more vascular rejection with arterial inflammation and a high prevalence of hypertension (Lefaucher et al. 2019).

Some data also indicate that these antibodies are implicated in the pathogenesis of focal and segmental glomerulosclerosis after kidney transplantation (Abuzeineh et al. 2020) AT1R antibodies were also prevalent in patients with hypertension (Philogene et al. 2020)], and these antibodies are considered to have some significance in the pathogenesis of primary aldosteronism (Sabbadin et al. 2018). These antibodies are also involved in the pathogenesis of preeclampsia (Zhou et al. 2008).

It was also found that AT1R antibodies have higher concentrations in patients with lupus nephritis than in a healthy control group (Xiong et al. 2013). Some published data indicate that 54.2% of patients with lupus nephritis have positive anti-AT1R antibodies. Moreover, anti-dsDNA (double-stranded deoxyribonucleic acid) antibodies, C3 and C4 serum levels were higher in positive anti-AT1R lupus patients. The cutoff point for a positive result was 17 U/ml in this study. Subintimal fibrosis and medial hyperplasia areas were also greater in the group that tested positive for anti-AT1R antibodies (Mejia-Vilet et al. 2020). Anti-AT1R antibodies were also earlier found in 66.29% of lupus nephritis patients, but no cutoff point was revealed in this study (Xiong et al. 2013).

Impairment of the AT1R function may also cause changes similar to changes caused by antibodies. Clinical situations when reduced expression of AT1R is observed may indicate the possible area for the AT1R antibodies studies. Reduced expression of angiotensin II type 1 and 2 receptors was observed in arteries from nasal lesions in granulomatosis with polyangiitis patients, suggesting the existence of a down-regulation mechanism of these receptors (Dimitrijevic et al. 2011). Increased AT1R expression was observed in the arteries of patients with giant cell arteritis (Dimitrijevic et al. 2009). AT1R seems to influence chronic tubulointerstitial damage connected with reperfusion after renal ischaemia. AT1R knockout prevented post-reperfusion changes in mice (Fujita et al. 2021). AT1R is implicated in the pathogenesis of anti-glomerular basement membrane disease (Kinoshita et al. 2011), reflux nephropathy (Chertin et al. 2002), diabetic nephropathy (Ajdinovic et al. 2015), and acute kidney injury during sepsis (Leisman et al. 2021). A1166C polymorphism of AT1R was found to be a predictor of renal function decline in a healthy Chinese population (Lee et al. 2009). AT1R expression may be modulated by salt intake in the diet during anti-thymocyte serum nephritis (Suzuki et al. 2004).

Interestingly, AT1R was detected in mesangial cells and renin-producing kidney cells (Schrankl et al. 2021). AT1R stimulation takes part in the amplification of oxidative stress in renal mesangial cells (Akaishi et al. 2019). AT1R also influences antifibrotic signal transduction in renal and cardiac fibrosis models (Chow et al. 2019). Moreover, stimulation of AT1R on lymphocyte T mitigates renal fibrosis (Wen et al.^2019^). AT1R pathway influences glomerular repair in a mouse glomerulonephritis model (Hayashi et al. 2012). Inhibition of AT1R signalling associated with aberrant podocytes ameliorates albuminuria and has a protective effect on the glomerular filtration barrier (Inoue et al. 2019). It was proved that AT1R antibodies hamper the mRNA expression of slit diaphragm molecules in an animal model, which results in proteinuria (Suzuki et al. 2007). Such observations may suggest a potential influence of AT1R on the course of glomerular disease.

There were no published data about the levels of angiotensin II receptor 1 antibodies in primary glomerulonephritis: membranous glomerulonephritis, focal and segmental glomerulosclerosis, IgA nephropathy and mesangial proliferative non-IgA glomerulonephritis.

We planned to assess the levels of AT1R antibodies in the serum of patients with the above-mentioned types of glomerulonephritis and compare them to those found in patients with lupus nephritis and systemic vasculitis.

## Materials and Methods

We collected serum samples from 136 patients with different glomerulopathy types. These were the following: 80 patients with glomerulonephritis (18 patients with membranous glomerulonephritis, including 6 patients with positive anti-phospholipase A2 receptor antibodies and 12 patients with negative anti-phospholipase A2 receptor antibodies, 25 patients with focal and segmental glomerulosclerosis, 17 patients with lupus nephritis, 14 patients with IgA nephropathy, 6 patients with mesangial proliferative non-IgA glomerulonephritis) and 56 patients with systemic vasculitis, including 40 patients with c-ANCA (anti-neutrophil cytoplasmic antibodies) vasculitis and 16 patients with p-ANCA vasculitis. We obtained approval No. B-546/2012 to perform the study from the Bioethics Committee of Wrocław Medical University. All patients signed informed consent forms before taking part in the study. We collected material from consecutive patients who came to our clinic without any particular selection key. The study was performed without any special randomisation. The inclusion criteria consisted of proteinuria in the last available urine sample and known histopathological diagnosis. All patients were admitted to the hospital due to initial manifestation of the disease or relapse. Material was collected before starting intensive treatment (steroid pulse or cyclophosphamide). Exclusion criteria included renal replacement therapy, present or past malignancy, and active infection at the moment of material collection.

Blood collection was performed using additional tubes during a standard blood sampling for laboratory procedures, where 2.7 ml of blood was collected. Blood was collected between 8 and 9 a.m. Patients were not obliged to fast beforehand. The distance between the laboratory and the clinic is very short, so it was possible to transport the samples swiftly (less than10 min). The material was not collected when the temperature was higher than 28 ℃. Next, blood was centrifuged at 1500 g for 10 min at room temperature. Afterward, the obtained serum was frozen at – 80 °C.

The AT1R antibodies serum concentration was assessed using commercially available kits based on enzyme-linked immunosorbent assay (CellTrend, Luckenwalde, Germany), following the manufacturer’s instructions. The samples were evaluated on a precoated microtiter plate. Diluted 1:100 samples and standards were added into the wells and incubated for 2 h at 2–8 ℃. Next, the washing steps were followed. Afterward, non-HLA antibody detection with a POD (horseradish peroxidase)-labelled anti-human IgG antibody (1:100) was performed, followed by colour development with TMB (3, 3′, 5, 5′-tetramethylbenzidine) substrate solution measured at 450 nm. The correction wavelength was set at 630 nm. The conversion of optical density into concentration was performed using standard curves. The results > 10 U/ml were approved as positive.

Clinical data about basic creatinine, proteinuria, total protein, albumin, age, and sex of patients were collected.

Moreover, levels of creatinine, total protein, and albumin were noted 1 month, 3 months, 6 months, 12 months, 2 years, and 4 years after collection of material for AT1R antibodies analysis.

## Statistical Analysis

Analysis of the comparisons of AT1R antibodies and clinical data (proteinuria, age, serum creatinine, serum albumin, and serum total protein) between several groups of patients was performed using Tukey test. Analysis of the percentage of positive AT1R antibodies results between specific groups of patients was performed using a chi-square test.

Data were also examined using correlation analysis of quantitative variables. Most data do not have a normal distribution (Shapiro–Wilk test). Pearson’s correlation coefficient was applied when at least one specimen had a normal distribution. Spearman’s rank correlation coefficient was applied to analyse the data in the case of non-normal distribution in both specimens. The Spearman and Pearson correlation coefficients were examined with the *t* test to check their statistical significance. All statistical analyses were approved as positive when *p* < 0.05. The links between the following variables were tested: AT1R antibodies, serum creatinine, serum total protein, serum albumin, and proteinuria. In the case of lupus nephritis, the correlation between AT1R antibodies and antinuclear antibody (ANA) level was also tested. Correlations between AT1R antibody levels and c-ANCA levels in c-ANCA vasculitis and p-ANCA level in p-ANCA vasculitis were tested as well.

Moreover, analysis of creatinine, proteinuria, albumin, and total protein levels in prospective observation dependently on basic AT1R antibodies level was performed (hierarchic linear modelling).

## Results

The median and mean AT1R antibodies levels and comparison of mean AT1R antibodies levels between specific glomerular diseases (Tukey test) are presented in Table 1.

**Table 1:** Values of the median and mean AT1R antibodies levels and comparison of mean AT1R antibodies levels between specific glomerular diseases (Tukey test)

	Median AT1R antibodies levels (U/ml)	Mean AT1R antibodies (U/ml)	Membranous nephropathy	Focal and segmental glomerulosclerosis	Lupus nephritis	IgA nephropathy	Mesangial proliferative (non-IgA) glomerulonephritis	C-ANCA vasculitis	P-ANCA vasculitis
Membranous nephropathy	6 (range: 3–7.8)	5.67 ± 1.31	NA	NS	*p* ≤ 0.001	NS	NS	*p* ≤ 0.001	*p* ≤ 0.001
Focal and segmental glomerulosclerosis	5.6 (range: 3.8–14.7)	6.26 ± 2.25	NS	NA	*p* ≤ 0.001	NS	NS	*p* ≤ 0.001	*p* ≤ 0.001
Lupus nephritis	9.3 (range: 5.6–34.7)	10.6 ± 6.72	*p* ≤ 0.001	*p* ≤ 0.001	NA	*p* ≤ 0.01	NS	NS	NS
IgA nephropathy	5.5 (range: 4.2–11.7)	6.69 ± 2.52	NS	NS	*p* ≤ 0.01	NA	NS	*p* ≤ 0.001	*p* ≤ 0.001
Mesangial proliferative (non-IgA) glomerulonephritis	6.5 (range: 4.2–8.4)	6.53 ± 1.38	NS	NS	NS	NS	NA	*p* ≤ 0.01	*p* ≤ 0.001
C-ANCA vasculitis	7.35 (range: 3.9–51.9)	11.22 ± 10.78	*p* ≤ 0.001	*p* ≤ 0.001	NS	*p* ≤ 0.001	*p* ≤ 0.01	NA	NS
P-ANCA vasculitis	7.55 (range: 3.2–51.2)	12.65 ± 14.59	*p* ≤ 0.001	*p* ≤ 0.001	NS	*p* ≤ 0.001	*p* ≤ 0.001	NS	NA

*P* probability value (statistically important if *p* < 0.05), *NS*-*p* is not statistically significant, *NA* not applicable

The mean AT1R antibodies level in lupus nephritis was higher than in membranous nephropathy, focal and segmental glomerulosclerosis, IgA nephropathy. Moreover, the mean AT1R antibodies levels in c-ANCA vasculitis and p-ANCA vasculitis were higher than in membranous nephropathy, focal and segmental glomerulosclerosis, IgA nephropathy and mesangial proliferative (non-IgA) glomerulonephritis.

Mean AT1R antibodies in c-ANCA vasculitis and p-ANCA vasculitis were not statistically different from AT1R antibodies in lupus nephritis.

We also compared the percentage of patients with particular diseases with positive AT1R antibodies. We set the cutoff point for a positive result as > 10 U/ml. This level is one of the accepted cutoff points described in other studies (Banasik et al. 2014a; Sorohan et al. 2020).

The results are presented in Table 2. We found that 0–14% of patients with primary glomerulonephritis (dependently of the type of glomerulonephritis), 19–30% of patients with vasculitis (dependently of the type vasculitis) and 41% with lupus nephritis had AT1R antibodies higher than 10 U/ml. The percent of patients with positive AT1R antibodies in lupus nephritis was statistically significantly higher than in membranous nephropathy, focal and segmental glomerulosclerosis, IgA nephropathy, mesangial proliferative (non-IgA glomerulonephritis, p-ANCA vasculitis according to a chi-square test. In addition, c-ANCA vasculitis patients significantly more often had positive results than membranous nephropathy, focal and segmental glomerulosclerosis, IgA nephropathy, mesangial proliferative (non-IgA) glomerulonephritis. Positive results of p-ANCA vasculits patients occur more frequent than among membranous nephropathy, focal and segmental glomerulosclerosis, mesangial proliferative (non-IgA) glomerulonephritis patients.

**Table 2:** AT1R antibodies values in glomerular diseases compared to the range 10 U/ml and comparison of them using chi-square test

Glomerular disease	Number of patients	% of patients with AT1R antibodies < 10 U/ml	% of patients with AT1R antibodies > 10 U/ml	Membranous nephropathy	Focal and segmental glomerulosclerosis	Systemic lupus erythematosus	IgA nephropathy	Mesangial proliferative non-IgA glomerulonephritis	C-ANCA vasculitis	P-ANCA vasculitis
Membranous nephropathy	18	100	0	NA	*p* ≤ 0.05	*p* ≤ 0.001	*p* ≤ 0.001	NS	*p* ≤ 0.001	*p* ≤ 0.001
Focal and segmental glomerulosclerosis	25	96	4	*p* ≤ 0.05	NA	*p* ≤ 0.001	*p* ≤ 0.05	*p* ≤ 0.05	*p* ≤ 0.001	*p* ≤ 0.001
Systemic lupus erythematosus	17	59	41	*p* ≤ 0.001	*p* ≤ 0.001	NA	*p* ≤ 0.001	*p* ≤ 0.001	NS	*p* ≤ 0.001
IgA nephropathy	14	86	14	*p* ≤ 0.001	*p* ≤ 0.05	*p* ≤ 0.001	NA	*p* ≤ 0.001	*p* ≤ 0.01	NS
Mesangial proliferative non-IgA glomerulonephritis	6	100	0	NS	*p* ≤ 0.05	*p* ≤ 0.001	*p* ≤ 0.001	NA	*p* ≤ 0.001	*p* ≤ 0.001
C-ANCA vasculitis	40	70	30	*p* ≤ 0.001	*p* ≤ 0.001	NS	*p* ≤ 0.01	*p* ≤ 0.001	NA	NS
P-ANCA vasculitis	16	81	19	*p* ≤ 0.001	*p* ≤ 0.001	*p* ≤ 0.001	NS	*p* ≤ 0.001	NS	NA

The correlation between the level of AT1R antibodies in lupus nephritis and ANA level in this group of patients was not statistically significant. The correlation between AT1R antibodies and c-ANCA levels in c-ANCA vasculitis and the correlation between AT1R antibodies and p-ANCA level in p-ANCA vasculitis were also not statistically significant.

Clinical parameters means of patients groups with specific glomerular diseases and comparisons between these means (Tukey test) are presented in Table 3.

**Table 3:** Clinical parameters means of patients groups with specific glomerular diseases and comparisons between these means (Tukey test)

Disease	Creatinine (mg/dl)	Proteinuria (g/24 h)	Total protein (g/dl)	Total albumin (g/dl)	Age (years)	Membranous nephropathy	Focal and segmental glomerulosclerosis	Lupus nephritis	IgA nephropathy	Mesangial proliferative (non-IgA glomerulonephritis)	C-ANCA vasculitis	P-ANCA vasculitis
Membranous nephropathy	1.37 ± 0.57	4.23 ± 4.58	4.81 ± 0.70	2.79 ± 0.64	52.00 ± 12.00	NA	Creatinine: NS Proteinuria NS Total protein: *p* ≤ 0.001 Total albumin: NS Age: *p* ≤ 0.05	Creatinine: NS Proteinuria: *p* ≤ 0.001 Total protein: *p* ≤ 0.001 Total albumin: *p* ≤ 0.05 Age: *p* ≤ 0.001	Creatinine: NS Proteinuria: *p* ≤ 0.001 Total protein: *p* ≤ 0.001 Total albumin: *p* ≤ 0.001 Age: *p* ≤ 0.001	Creatinine: NS Proteinuria NS Total protein: NS Total albumin: NS Age: *p* ≤ 0.001	Creatinine: NS Proteinuria: *p* ≤ 0.001 Total protein: *p* ≤ 0.001 Total albumin: *p* ≤ 0.001 Age: NS	Creatinine: *p* ≤ 0.001 Proteinuria: *p* ≤ 0.001 Total protein: *p* ≤ 0.001 Total albumin: ≤ 0.001 Age: *p* ≤ 0.001
Focal and segmental glomerulosclerosis	1.32 ± 0.53	3.17 ± 3.11	5.20 ± 1.16	2.99 ± 0.91	46.36 ± 15.00	Creatinine: NS Proteinuria: NS Total protein: *p* ≤ 0.001 Total albumin: NS Age: *p* ≤ 0.05	NA	Creatinine: NS Proteinuria: *p* ≤ 0.01 Total protein: *p* ≤ 0.05 Total albumin: NS Age: *p* ≤ 0.001	Creatinine: NS Proteinuria: *p* ≤ 0.001 Total protein: NS Total albumin: *p* ≤ 0.05 Age: NS	Creatinine: NS Proteinuria: NS Total protein: NS Total albumin: NS Age: *p* ≤ 0.001	Creatinine: NS Proteinuria: *p* ≤ 0.001 Total protein: *p* ≤ 0.001 Total albumin: *p* ≤ 0.001 Age: *p* ≤ 0.001	Creatinine: *p* ≤ 0.001 Proteinuria: NS Total protein: *p* ≤ 0.001 Total albumin: *p* ≤ 0.001 Age: *p* ≤ 0.001
Lupus nephritis	1.21 ± 0.42	1.85 ± 1.70	5.60 ± 0.84	3.18 ± 0.59	37.82 ± 13.00	Creatinine: NS Proteinuria: *p* ≤ 0.001 Total protein: *p* ≤ 0.001 Total albumin: *p* ≤ 0.05 Age *p* ≤ 0.001	Creatinine: NS Proteinuria: *p* ≤ 0.01 Total protein: *p* ≤ 0.05 Total albumin: NS Age *p* ≤ 0.001	NA	Creatinine: NS Proteinuria: NS Total protein: NS Total albumin: NS Age *p* ≤ 0.05	Creatinine: NS Proteinuria: NS Total protein: NS Total albumin: NS Age: NS	Creatinine: *p* ≤ 0.05 Proteinuria: NS Total protein: *p* ≤ 0.01 Total albumin: *p* ≤ 0.001 Age *p* ≤ 0.001	Creatinine: *p* ≤ 0.001 Proteinuria: NS Total protein: NS Total albumin: NS Age *p* ≤ 0.001
IgA nephropathy	1.19 ± 0.34	1.44 ± 1.29	5.57 ± 0.77	3.32 ± 0.64	43.86 ± 15.00	Creatinine: NS Proteinuria: *p* ≤ 0.001 Total protein: *p* ≤ 0.001 Total albumin: *p* ≤ 0.001 Age *p* ≤ 0.001	Creatinine: NS Proteinuria: *p* ≤ 0.001 Total protein: NS Total albumin: *p* ≤ 0.05 Age: NS	Creatinine: NS Proteinuria: NS Total protein: NS Total albumin: NS Age *p* ≤ 0.05	NA	Creatinine: NS Proteinuria: *p* ≤ 0.05 Total protein: NS Total albumin: NS Age *p* ≤ 0.001	Creatinine: *p* ≤ 0.01 Proteinuria: NS Total protein: *p* ≤ 0.01 Total albumin: NS Age *p* ≤ 0.001	Creatinine: *p* ≤ 0.001 Proteinuria: NS Total protein: *p* ≤ 0.05 Total albumin: NS Age *p* ≤ 0.001
Mesangial proliferative (non-IgA) glomerulonephritis	1.01 ± 0.36	3.16 ± 2.54	4.68 ± 0.51	2.54 ± 0.61	31.50 ± 13.00	Creatinine: NS Proteinuria: NS Total protein: NS Total albumin: NS Age *p* ≤ 0.001	Creatinine: NS Proteinuria: NS Total protein: NS Total albumin: NS Age *p* ≤ 0.001	Creatinine: NS Proteinuria: NS Total protein: NS Total albumin: NS Age NS	Creatinine: NS Proteinuria: *p* ≤ 0.05 Total protein: NS Total albumin: NS Age *p* ≤ 0.001	NA	Creatinine: *p* ≤ 0.01 Proteinuria: NS Total protein: *p* ≤ 0.001 Total albumin: NS Age *p* ≤ 0.001	Creatinine: *p* ≤ 0.001 Proteinuria: NS Total protein: *p* ≤ 0.001 Total albumin: NS Age *p* ≤ 0.001
c-ANCA vasculitis	2.07 ± 1.54	1.72 ± 3.61	6.29 ± 0.44	3.63 ± 0.46	55.00 ± 18.00	Creatinine: NS Proteinuria: *p* ≤ 0.001 Total protein: *p* ≤ 0.001 Total albumin: *p* ≤ 0.001 Age: NS	Creatinine: NS Proteinuria: *p* ≤ 0.001 Total protein: *p* ≤ 0.001 Total albumin: *p* ≤ 0.001 Age *p* ≤ 0.001	Creatinine: *p* ≤ 0.05 Proteinuria: NS Total protein: *p* ≤ 0.01 Total albumin: *p* ≤ 0.001 Age *p* ≤ 0.001	Creatinine: *p* ≤ 0.01 Proteinuria: NS Total protein: *p* ≤ 0.01 Total albumin: NS Age *p* ≤ 0.001	Creatinine: *p* ≤ 0.01 Proteinuria: NS Total protein: *p* ≤ 0.001 Total albumin: *p* ≤ 0.05 Age *p* ≤ 0.001	NA	Creatinine: *p* ≤ 0.001 Proteinuria: NS Total protein: NS Total albumin: NS Age *p* ≤ 0.001
p-ANCA vasculitis	3.93 ± 2.39	2.46 ± 3.08	6.09 ± 0.85	3.53 ± 0.42	62.00 ± 12.00	Creatinine: *p* ≤ 0.001 Proteinuria: *p* ≤ 0.001 Total protein: *p* ≤ 0.001 Total albumin: *p* ≤ 0.001 Age *p* ≤ 0.001	Creatinine: *p* ≤ 0.001 Proteinuria: NS Total protein: *p* ≤ 0.001 Total albumin: *p* ≤ 0.001 Age *p* ≤ 0.001	Creatinine: *p* ≤ 0.001 Proteinuria: NS Total protein: NS Total albumin: NS Age *p* ≤ 0.001	Creatinine: *p* ≤ 0.001 Proteinuria: NS Total protein: *p* ≤ 0.05 Total albumin: NS Age *p* ≤ 0.001	Creatinine: *p* ≤ 0.001 Proteinuria: NS Total protein: *p* ≤ 0.001 Total albumin: NS Age *p* ≤ 0.001	Creatinine: *p* ≤ 0.001 Proteinuria: NS Total protein: NS Total albumin: NS Age *p* ≤ 0.001	NA

*P* probability value (statistically important if *p* < 0.05); *NS*-*p* is not statistically significant; *NA* not applicable

Clinical data comparison indicated that the creatinine level was not statistically difference between lupus nephropathy and membranous nephropathy, focal and segmental glomerulosclerosis, IgA nephropathy, mesangial proliferative (non-IgA) glomerulonephritis.

Creatinine level in c-ANCA vasculitis was higher compared to creatinine level in lupus nephritis, IgA nephropathy and mesangial proliferative (non-IgA) glomerulonephritis, but lower compared to p-ANCA vasculitis.

Creatinine level in p-ANCA vasculitis was higher compared to all other groups.

Proteinuria in patients with membranous nephropathy was higher than among patients with lupus nephritis, IgA nephropathy, c-ANCA vasculitis and p-ANCA vasculitis.

Proteinuria in patients with focal and segmental glomerulosclerosis was higher than proteinuria in lupus nephritis, c-ANCA vasculitis, and p-ANCA vasculitis patients.

Proteinuria in lupus nephritis group was lower compared to membranous nephropathy and focal and segmental glomerulosclerosis groups.

Proteinuria in IgA nephropathy group was lower compared to membranous nephropathy, focal and segmental glomerulosclerosis and mesangial proliferative (non-IgA) glomerulonephritis.

Proteinuria in patients with mesangial proliferative glomerulonephritis was higher compared to IgA nephropathy group.

c-ANCA vasculitis group proteinuria was lower compared to membranous nephropathy and focal and segmental glomerulosclerosis groups.

p-ANCA vasculitis group proteinuria was lower compared to membranous nephropathy group.

Total protein level in the membranous nephropathy group was lower compared to the total protein level in other groups apart mesangial proliferative glomerulonephritis.

Total serum protein concentration was also lower in the focal and segmental glomerulosclerosis groups compared to lupus nephritis, c-ANCA, and p-ANCA vasculitis and higher compared to membranous nephropathy.

Lupus nephritis group total protein level was lower compared to c-ANCA vasculitis group and higher compared to membranous nephropathy and focal and segmental glomerulosclerosis.

IgA nephropathy group and mesangial proliferative glomerulonephritis group total protein levels were lower compared to c-ANCA and p-ANCA vasculitis groups. IgA nephropathy total protein level was higher compared to membranous nephropathy group.

c-ANCA vasculitis total protein level was higher compared to membranous nephropathy, focal and segmental glomerulosclerosis, lupus nephritis, IgA nephropathy and mesangial proliferative (non-IgA) glomerulonephritis.

p-ANCA vasculitis total protein level was higher compared to membranous nephropathy, focal and segmental glomerulosclerosis, IgA nephropathy and mesangial proliferative (non-IgA) glomerulonephritis.

Albumin level in the membranous nephropathy group was lower in comparison with IgA nephropathy, lupus nephritis, c-ANCA, and p-ANCA vasculitis groups.

Albumin level in the focal and segmental glomerulosclerosis group was lower compared to IgA nephropathy, c-ANCA, and p-ANCA vasculitis groups.

Albumin level in the lupus nephritis group was higher compared to membranous nephropathy group and lower compared to c-ANCA vasculitis group.

Albumin level in the IgA nephropathy group was higher compared to membranous nephropathy and focal and segmental glomerulosclerosis groups.

Albumin level in the mesangial proliferative (non-IgA) glomerulonephritis do not differ significantly from the other groups.

Albumin level in c-ANCA vasculitis group was higher than this level in the membranous nephropathy, focal and segmental glomerulosclerosis, lupus nephritis and mesangial proliferative (non-IgA) glomerulonephritis groups.

Albumin level in p-ANCA vasculitis group was higher compared to membranous nephropathy, focal and segmental glomerulosclerosis and mesangial proliferative (non-IgA) glomerulonephritis groups.

Membranous nephropathy patients were older than focal and segmental glomerulosclerosis, IgA nephropathy, lupus nephritis, mesangial proliferative (non-IgA) glomerulonephritis patients, but younger compared with p-ANCA vasculitis patients.

Age of focal and segmental glomerulosclerosis patients was higher compared to lupus nephritis and mesangial proliferative (non-IgA) glomerulonephritis, but lower than the age of membranous nephropathy, c-ANCA and p-ANCA vasculitis patients.

Lupus nephritis patients were younger compared to membranous nephropathy, focal and segmental glomerulosclerosis, IgA nephropathy, c-ANCA, and p-ANCA vasculitis patients.

IgA nephropathy patients were older than lupus nephritis and mesangial proliferative (non-IgA) glomerulonephritis patients, but younger compared to membranous nephropathy, c-ANCA and p-ANCA vasculitis patients.

Mesangial proliferative (non-IgA) glomerulonephritis patients were younger compared to all groups apart lupus nephritis group.

c-ANCA vasculitis patients were older compared to focal and segmental glomerulosclerosis, lupus nephritis, IgA nephropathy and mesangial proliferative (non-IgA) glomerulonephritis, but younger compared to p-ANCA vasculitis.

p-ANCA vasculitis patients were older compared to all other groups.

There was no statistically significant correlation between AT1R antibodies and creatinine level in any patient group. The correlations between proteinuria and AT1R antibodies levels were also statistically insignificant. A significant inverse correlation between AT1R antibodies and albumin level in the membranous nephropathy group, *r* =  − 0.51 (*p* ≤ 0.05), was found. Interestingly, in membranous nephropathy patients, the correlation between proteinuria and AT1R antibodies was very close to statistical significance, *r* = 0.42 (*p* = 0.08).Simultaneously, an inverse correlation between total protein serum level and AT1R antibodies, *r* =  − 0.42 (*p* = 0.07), was also very close to statistical significance. Overall, AT1R antibodies appear to have some connection with membranous nephropathy activity (probably in the case of a larger patient group, these correlations would be statistically significant). However, this connection does not appear to be very strong. There was no statistical difference in AT1R antibodies between PLA2R (anti-phospholipase A2 receptor antibody) positive and PLA2R negative patients (*t* test).

Prospective analysis of total protein level reveals increasing levels of total protein during time among patients with higher AT1R antibodies levels in focal and segmental glomerulosclerosis, lupus nephritis, IgA nephropathy, c-ANCA and p-ANCA vasculitis.

Prospective analysis of albumin levels showed increasing levels of albumin during time among patients with higher AT1R antibodies levels in lupus nephritis, IgA nephropathy, c-ANCA and p-ANCA vasculitis.

Prospective analysis of creatinine levels indicated an increase of creatinine levels during time among patients with higher AT1R antibodies levels in p-ANCA vasculitis.

## Discussion

Systemic lupus erythematosus (Koffler et al. 1967) and systemic vasculitis (Shillitoe et al. 1974) are active systemic diseases. Primary glomerulonephritis is predominantly a local disease involving the kidney with some additional systemic involvement markers such as anti-PLAR2 antibodies in membranous nephropathy (Beck et al. 2009), IgA1 in IgA nephropathy (Moldoveanu et al. 2007). We checked AT1R antibodies levels in serum, so we supposed that immunologically active systemic diseases such as lupus nephritis and vasculitis should be related to higher antibodies levels compared to primary glomerulonephritis. AT1R antibodies evaluation confirmed our suspicions. Lupus nephritis and vasculitis patients have higher AT1R antibodies levels than membranous nephropathy, focal and segmental glomerulosclerosis, IgA nephropathy, and mesangial proliferative (non-IgA) glomerulonephritis patients. We were the first to note that primary glomerulonephritis is connected with relatively low AT1R antibodies levels with a very high percentage (86–100%) of patients with levels lower than the cutoff point for a positive result. Nevertheless, an inverse correlation between AT1R antibodies and serum albumin levels in membranous nephropathy indicates that despite low values, AT1R antibodies may have some clinical significance even in the early stage of membranous glomerulonephritis. Our membranous nephropathy results are consistent with a previous observation that AT1R may influence the glomerular filtration barrier (Inoue et al. 2019).

There is of course some problem in this study with the heterogeneity of the groups with some differences in basic clinical parameters, but statistically important correlations between these parameters and AT1R antibodies levels were not found. Moreover, patients in these studies were consequent patients admitted to the clinic without any exclusion apart exclusion criteria.

Taking into account such kind of inclusion to the study, the clinical parameters of the patients in specific groups represent typical parameters appeared in real clinical patients and not perfect groups of patients with all same parameters but created by artificial selection.

Prospective analysis of total protein, albumin, creatinine dependently of basic AT1R antibodies levels gave no answer about the real influence of AT1R antibodies on the course of the disease. Most of the patients groups present an increase of total protein and albumin during time connected with higher basic AT1R antibodies levels. This influence is not possible to distinguish from the effect of patient treatment. Patients with active disease probably were more intensively treated, so the increase of total protein and albumin may reflect the efficacy or intensivity of treatment.

Higher creatinine level during time in patients with higher AT1R antibodies levels in p-ANCA group may be really connected with the immunological activity of the disease, but the influence of natural progress of chronic kidney disease on these results is difficult to exclude. p-ANCA vasculits group had the highest creatinine levels from all groups.

The results of this study indicate that AT1R antibodies appear in many patients with lupus nephropathy and vasculitis before end-stage kidney disease. Future studies evaluating the impact of early detected AT1R antibodies on the course of disease, including what happens after kidney transplantation, should be considered. At present, it is only known that pretransplant AT1R antibodies predict graft function and risk of allograft rejection in patients with low-risk kidney transplantation. The pretransplant assessment of AT1R antibodies in this study was performed in samples collected shortly before transplantation (Yu et al. 2020). It would be interesting to compare kidney transplant rejection rate and survival between patients with primary glomerulonephritis with higher versus lower levels of AT1R antibodies from the onset of the disease.

## Conclusions

The mean AT1R antibodies level in lupus nephritis was higher than in membranous nephropathy, focal and segmental glomerulosclerosis, IgA nephropathy. Moreover, the mean AT1R antibody levels in c-ANCA vasculitis and p-ANCA vasculitis were higher than in membranous nephropathy, focal and segmental glomerulosclerosis, IgA nephropathy and mesangial proliferative (non-IgA) glomerulonephritis. AT1R antibodies are inversely correlated with serum albumin during membranous nephropathy. Prospective analysis of creatinine levels indicated an increase of creatinine levels during time among patients with higher AT1R antibodies levels in p-ANCA vasculitis.
